# Upward Effortful Pitch Glide as a Predictor of Pharyngeal Constriction in Individuals With Upper Versus Lower Motor Neuron Lesions

**DOI:** 10.1044/2026_AJSLP-25-00452

**Published:** 2026-08-20

**Authors:** Scott King, Elizabeth Lanza, Benjamin Schiedermayer, Zhining Ou, Julie Barkmeier-Kraemer

**Affiliations:** a Department of Otolaryngology—Head & Neck Surgery, University of Utah Health, Salt Lake City; b Spencer Fox Eccles School of Medicine, University of Utah Health, Salt Lake City; c Utah Voice Disorders Center, University of Utah Health, Salt Lake City; d Center for Airway, Voice & Swallowing, UC San Diego Health, CA; e Department of Internal Medicine, Division of Epidemiology, University of Utah Health, Salt Lake City; f Department of Communication Sciences and Disorders, College of Health, University of Utah, Salt Lake City

## Abstract

**Purpose:**

This study tested the validity of using nasoendoscopy ratings of volitional maximum pharyngeal constriction (PCmax) during nasoendoscopy recordings of effortful upward pitch glide to predict pharyngeal area at maximum constriction (PAM) during swallowing recordings (VFSSs) in individuals with upper motor neuron (UMN) versus lower motor neuron (LMN) lesions.

**Method:**

Retrospective analysis of electronic medical records identified individuals with UMN or LMN lesions who underwent both VFSS and nasoendoscopy within 3 months. Blinded expert judges rated the degree and symmetry of PCmax in paired nasoendoscopic images of quiet breathing (minimum pharyngeal constriction) and highest pitch production (PCmax). Sixty-five percent of paired images were rated twice for intra- and interrater reliability estimates of agreement; consensus-based ratings were used when disagreement occurred. Comparisons between PCmax ratings and dichotomized VFSS PAM measures from 3-cc and 20-cc liquid bolus trials within UMN and LMN groups were assessed using univariate logistic regression.

**Results:**

Forty-six individuals (*N* = 24 LMN, 22 UMN) met inclusion criteria. Judge 1 achieved an intraclass correlation coefficient of .76, while Judge 2 achieved .71 intrarater agreement on PCmax measures; interrater agreement was .73 prior to reaching consensus. Nasoendoscopic ratings of PCmax did not differentiate “normal” and “abnormal” VFSS PAM measures in the UMN group (*p* = .89). In contrast, the odds ratio for correspondence between high PCmax scores (i.e., greater pharyngeal constriction) and normal PAM measures was 1.91 (95% confidence interval [1.14, 4.52]; *p* = .053) in the LMN group.

**Conclusion:**

Those with LMN lesions show predictable correspondence between PCmax and PAM outcomes, while the PCmax measures of a volitional task may not predict PAM in UMN lesioned individuals.

The pharynx contributes significantly to multiple functions of the upper aerodigestive tract during respiration, speech/resonance, and deglutition (Choi et al., 2014; Shaker & Hogan, 2003). Assessing pharyngeal integrity and function is a component of both voice and swallowing assessments conducted by otolaryngologists and speech-language pathologists (SLPs). Depending on the primary clinical concern, different instrumental methods are employed. For suspected dysphagia, instrumental swallow evaluations such as videofluoroscopic swallowing study (VFSS) or flexible endoscopic evaluation of swallowing (FEES) are recommended to directly observe pharyngeal constrictor function (Giraldo-Cadavid et al., 2017; Martin-Harris et al., 2020; Miller et al., 2020). VFSS instrumental assessment enables observation of pharyngeal constrictor function across all domains of deglutition. FEES offers direct observation of the pharyngeal region before and after the swallow. This enables assessment of symmetry of muscle tone and structural integrity in addition to visual perceptual impressions regarding the degree and duration of “white out” during the swallow that, combined with postswallow signs of bolus clearance and entry into the subglottal regions, can inform whether additional radiological testing is needed to further test oropharyngeal pathophysiology using VFSS. In contrast, an individual presenting with voice-related concerns typically undergoes endoscopic voice evaluation using either a nasoendoscope or a transoral rigid endoscope (Chandrasekhar et al., 2013; Patel et al., 2018), where swallowing anatomy and physiology may be screened but FEES or VFSS may not be recommended unless signs or symptoms of dysphagia are indicated.

Within the context of an endoscopic voice assessment, the upward effortful pitch glide (EPG; Malandraki et al., 2011) and the pharyngeal squeeze maneuver (PSM; Miles & Hunting, 2023) have been recommended as clinical screening tasks to evaluate pharyngeal constrictor activation using nasoendoscopy. The EPG is a volitional voicing task in which the patient sustains phonation while gliding from a low or comfortable pitch to their highest possible pitch, typically on the vowel /i/ (Malandraki et al., 2011; Miloro et al., 2014; Mira et al., 2024). In contrast, individuals performing the PSM are instructed to sustain a shrill high-pitched phonation (Miles & Hunting, 2023).

Both maneuvers are shown to elicit bilateral “squeeze” or contraction of the pharyngeal constrictor muscles as a test of integrity and function, as displayed in Figures 1 and 2 (Malandraki et al., 2011; Miloro et al., 2014; Mira et al., 2024). Prior studies have suggested that the presence and degree of bilateral pharyngeal constriction observed during EPG or PSM correspond well with pharyngeal function during swallowing (Fuller et al., 2009; Malandraki et al., 2011). In general, EPG or PSM are both useful maneuvers for assessing cranial nerve (CN) function during nasoendoscopic imaging of the upper aerodigestive tract. Specifically, the pitch glide and production of a high pitch engages the bilateral extrinsic and intrinsic musculature of the larynx, enabling observation of symmetry and degree of vocal fold elongation during activation of the cricothyroid muscle (external branch of the superior laryngeal nerve, CN X), onset and maintenance of vocal fold adduction during phonation (recurrent laryngeal nerve, CN X), and elevation of the larynx via extrinsic laryngeal musculature (CN X/XII, V, and VII). In addition, bilateral activation of the pharyngeal constrictor muscles (CN IX/X) and soft palate muscles (CNs V and X) are directly observed during both pharyngeal maneuvers. However, these specific maneuvers may not directly translate between volitional voice production and swallowing function associated with the nonvolitional, patterned response of the oropharyngeal swallow that is triggered at the level of the brainstem (Kennedy et al., 2020).

**Figure 1.**
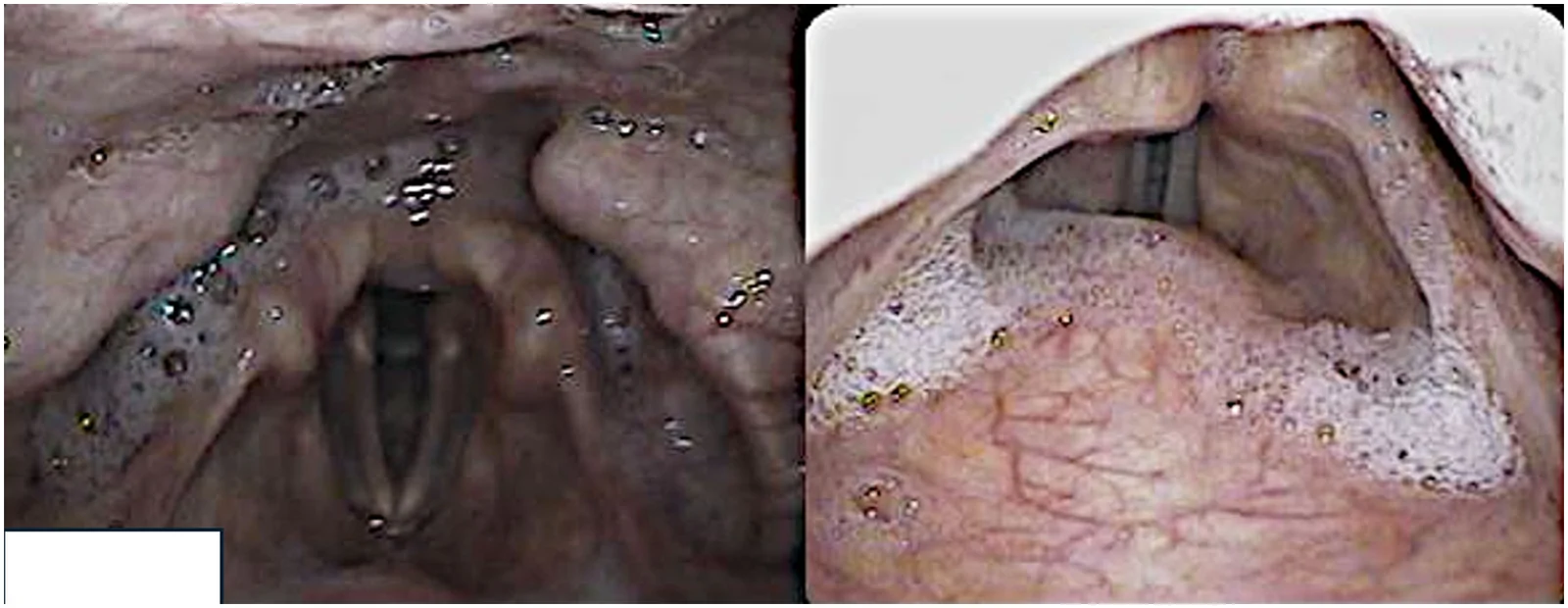
Example of typical nasoendoscopic views of the larynx and pharyngeal walls during rest breathing prior to onset of phonation (left panel) and at maximum pharyngeal constriction during production of the highest pitch (right panel). Note the vertically elevated position of the larynx in the right panel during the production of the highest pitch compared to the quiet breathing position in the left panel. The right panel exhibits bilateral pharyngeal constrictor activation that partially occludes views of the larynx and reduces the diameter of the pharynx.

**Figure 2.**
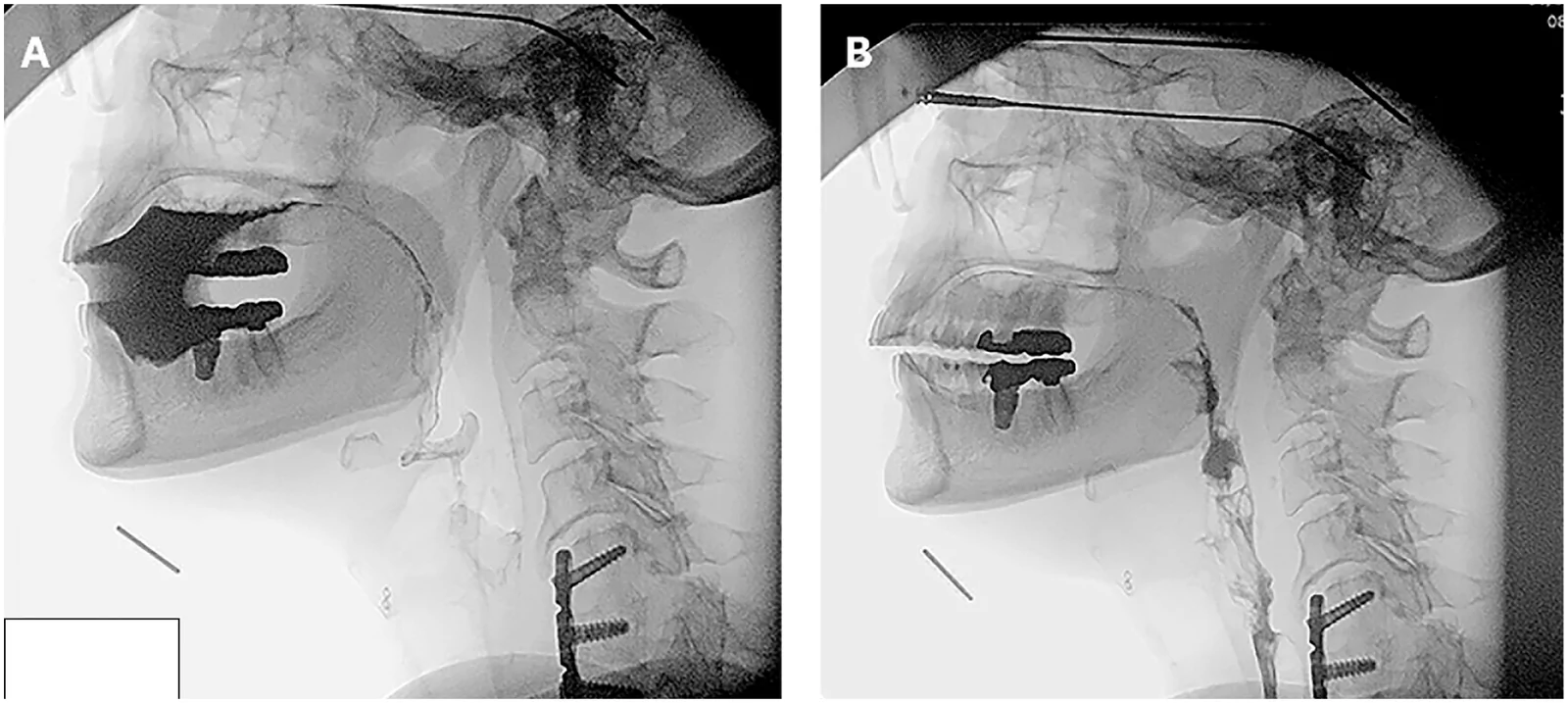
This figure displays videofluoroscopic swallowing study still images comparing pre-swallow and maximum pharyngeal constriction during a swallow in a representative lower motor neuron patient in the lateral plane of view. Panel A shows the patient while holding a 20-cc bolus within the oral cavity prior to initiation of the swallow. Panel B illustrates pharyngeal area at maximum constriction (PAM) during the first 20-cc liquid bolus swallow.

## Volitional Versus Nonvolitional Deglutition Pathways

Deglutition is a complex transitory process that is controlled by both volitional cortical input and nonvolitional brainstem circuitry (Jean, 2001; Lowell et al., 2008). The process of deglutition occurs on a continuum that has traditionally been described by phases or stages of deglutition to characterize the complex transitional physiology across participating anatomical regions including oral, pharyngeal, and esophageal domains. For the purpose of this study, we conceptually organize the voluntary or volitional elements of deglutition across the oral and initial portion of the pharyngeal domains of bolus preparation and transit as governed by cortical and subcortical upper motor neuron (UMN) circuitry (Cheng et al., 2022; Kamarunas et al., 2018; Ludlow, 2005, 2015; Ma et al., 2024; Wei et al., 2024; Wilmskoetter et al., 2019, 2020). In contrast, once triggered, the oropharyngeal swallow is considered a nonvolitional patterned response generating a well-organized and stereotypical motor sequence that propels the bolus through the pharynx and into the esophagus where a sequential peristaltic motor pattern continues involuntary transit of the bolus to the stomach. This triggered swallow pattern at the oropharyngeal domain is largely coordinated by brainstem-level central pattern generators that activate the neuromuscular pathways or lower motor neuron (LMN) pathways exiting the brainstem and cervical/thoracic spinal cord regions in coordination with respiration (Jean, 2001; Martin-Harris et al., 2003; Yagi et al., 2017). This distinction raises an important clinical question: Can pharyngeal constrictor function observed during volitional tasks such as EPG and PSM reliably predict constrictor function during the nonvolitional patterned response of the oropharyngeal swallow?

**Figure 3.**
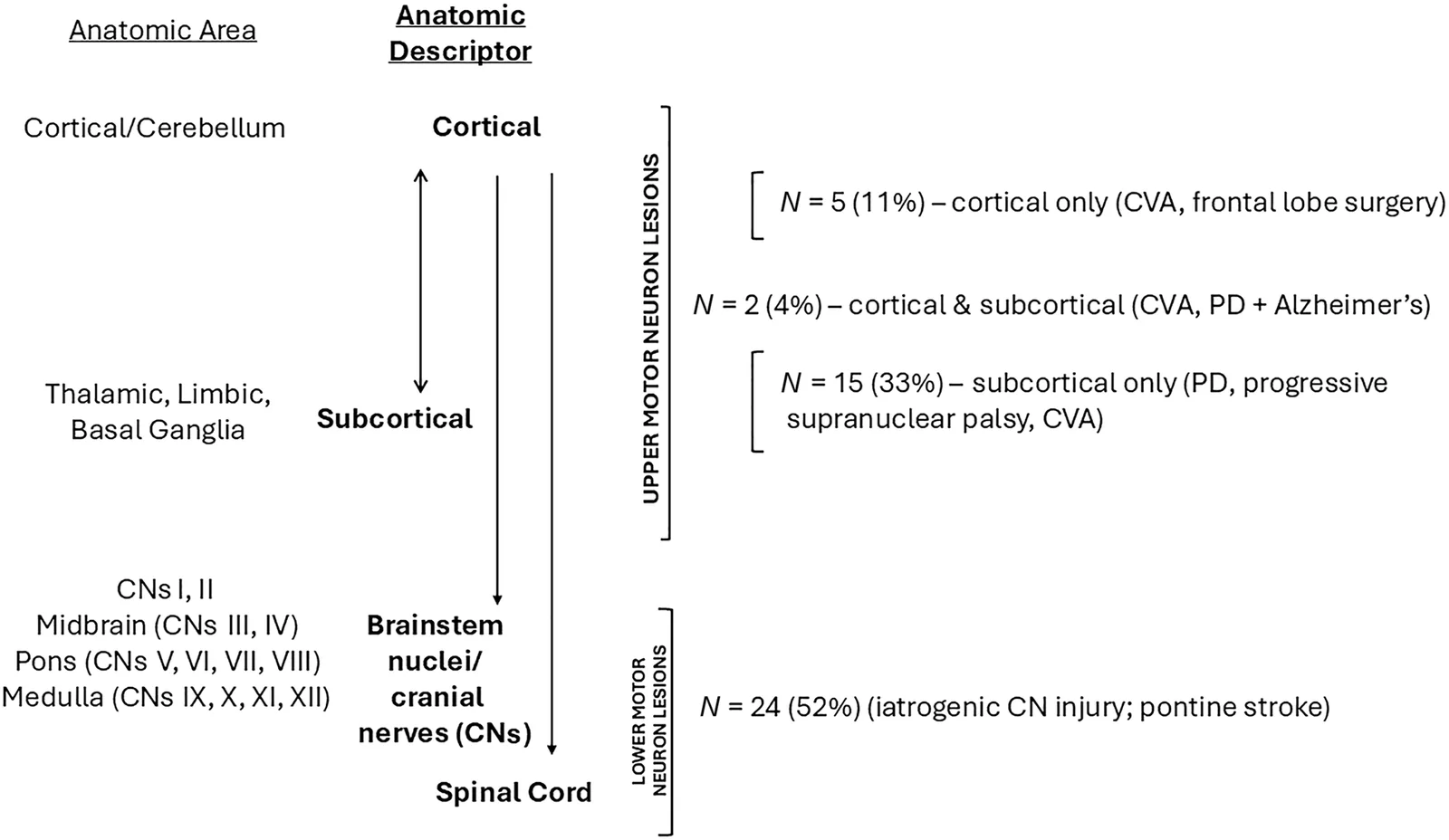
Participants for this study are aligned with the location of their lesion across upper motor neuron (UMN; cortical and subcortical lesions) and lower motor neuron (LMN; lesions between brainstem-level cell bodies and muscle end organ (i.e., peripheral nervous system). UMN lesions: lesions to neurons originating in the motor cortex that descend to the cell bodies of the brainstem or spinal cord nuclei (i.e., central nervous system [CNS]). LMN lesions: lesions to neurons located in the brainstem nuclei (CN) or anterior horn of the spinal cord that leave the CNS and innervate muscle fibers. CVA = cerebrovascular accident; PD = Parkinson's disease.

The answer may depend on the underlying neurological etiology of those being tested. In individuals with LMN lesions, impaired muscle contraction is typically observed across both volitional and nonvolitional tasks due to direct disruption of the final common pathway to the muscle (Ramahi et al., 2014; Stevens et al., 2022). In contrast, UMN lesions often impair volitional motor planning and execution while sparing brainstem-level patterned response neural pathways, leading to variability between volitional and nonvolitional behaviors (see Figure 3; Daniels et al., 2007; Kamarunas et al., 2018; Lowell et al., 2008, 2012; Steinhagen et al., 2009). Thus, while EPG and PSM may serve as a reliable predictor of pharyngeal constrictor function in LMN lesions, its predictive validity in UMN lesions remains uncertain.

## UMN Lesions and Dysphagia

Dysphagia characteristics have been described across multiple neurological populations associated with UMN impairment such as Parkinson's disease (PD), supratentorial-related stroke, and multiple sclerosis, among other examples (Martino et al., 2005; Pflug et al., 2018; Stathopoulos & Dalakas, 2022; Thomas & Wiles, 1999; Wilmskoetter et al., 2020). A signature feature of UMN damage or impairment described in these populations includes impaired planning, initiation, or volitional execution of motor behaviors resulting in variable behavior patterns, depending on the location, connectivity, and severity of the lesion (Daniels et al., 1999; Robbins & Levine, 1988; Robbins et al., 1993).

## LMN Lesions and Dysphagia

Dysphagia has been described in several populations with LMN lesions, such as spinal muscular atrophy (McGrattan et al., 2021), and idiopathic or iatrogenic postoperative onset of CN impairment (i.e., vocal fold paralysis; Schiedermayer et al., 2020; Stevens et al., 2022). In these populations, affected individuals exhibit muscle atrophy, fasciculations, and impaired muscle contraction resulting in observable impaired movement of affected structures. Thus, an LMN injury that directly or indirectly affects pharyngeal constrictor function would be characterized by absent or reduced constriction during swallowing or vocal activities that normally elicit muscular contraction. Signs of impaired function in these individuals would be indicated by incomplete clearance of boluses after the swallow, leaving bolus residue in the vallecular and/or pyriform sinuses and, in some cases, impaired airway safety (Stevens et al., 2022; Stokely et al., 2015; Waito et al., 2018). Due to the injured neural pathway that originates at the level of cell bodies within the brainstem or spinal cord and ending at the innervation site of the muscle fiber end organ, impaired muscle contraction is typically observed on the same side as the impaired neuromuscular pathway. Due to the weakened or absent signal from the CNS, impaired function would be observed during both volitional and nonvolitional domains of deglutition.

## Purpose of the Study

The current gap in the literature regarding the validity of volitional EPG for screening nonvolitional patterned swallowing responses involving the pharyngeal constrictor muscles has direct implications for clinical practice. If EPG reliably predicts swallowing function in LMN lesions but not UMN lesions, clinicians must exercise caution when generalizing findings from clinical voice and upper airway assessments to swallowing outcomes in neurologically diverse populations. The current study evaluated whether nasoendoscopic ratings of observed pharyngeal constriction during EPG corresponded to VFSS measures of maximum pharyngeal constriction (PCmax) in individuals with UMN versus LMN lesions. We hypothesize that EPG ratings will show stronger correspondence with VFSS outcomes in LMN lesions, whereas reduced correspondence will be observed in UMN lesions due to the differential involvement of volitional versus nonvolitional neural control mechanisms.

## Method

### Participants

This mixed methods study was approved by The University of Utah Institutional Review Board (Protocol #00117659). The protocol included an approved waiver of informed consent as all data were obtained retrospectively from medical records involving standard of care methodology. In addition, all patients at the University of Utah Health Voice Disorders Center signed a clinical consent form for the use of clinical recordings for research and educational purposes; therefore, all participants consented to the use of media accessed for this project. Protected health information was not associated with example images used in this article.

Individuals 18 years of age and older were included in this study and were previously evaluated in the University of Utah Health Voice Disorders Center between January 1, 2012, and January 1, 2023. Participants were included if they underwent both a VFSS and a nasoendoscopy exam that included an EPG task, within 3 months of each other, and without a change of medical or physical status between procedures. The latter was confirmed by inspecting patient-based report outcome questionnaires administered prior to each instrumental assessment consisting of the Dysphagia Handicap Index (Silbergleit et al., 2012) or the 10-item Eating Assessment Tool (Belafsky et al., 2008; depending on the year), and Voice Handicap Index (Jacobson et al., 1997). In addition, a review of the EMR was conducted to identify documentation of any new morbidities or changes to the participant's condition from the first to the second instrumental assessment that would exclude them from this study. Medical records were also inspected to classify individuals as belonging to either the UMN or LMN groups based on etiology or the most recent medical history associated with their referral for assessment. For this study, individuals with a singular diagnosis readily classified as a UMN (e.g., stroke, PD) or LMN (e.g., vocal fold paralysis) attributed to their onset of dysphagia were included. Those with multiple comorbidities that could not be classified as UMN or LMN were excluded.

### Nasoendoscopy Image Acquisition and Extraction

All nasoendoscopic images were acquired using one of the Clinic's PentaxMedical Laryngeal Imaging Systems during the time period under study. Most recent images for this study used the Pentax Medical Nasolaryngoscope System (KayPentax, Model 9310HD) to simultaneously capture audio and upper airway dynamic images, including the pharynx during clinical voice or swallowing assessment using nasoendoscopy (KayPentax, VNL-1070STK). A standard clinical voice assessment at the site under study instructs patients to perform an upward pitch glide to the maximal pitch that can be sustained for 1 s without screeching. The patient is typically elicited to repeat this task two to three times until the clinician is satisfied that they have performed a maximal high pitch. In most cases, clinicians offer a vocal demonstration of the task and coach the patient to achieve a higher pitch on each subsequent trial to assure the highest pitch possible. Clinicians also observe and document if patients exhibit secondary signs of struggle simultaneous with observations of upper airway structures to guide clinical decision making regarding the number of trials needed to satisfy a maximum pitch was achieved.

Participant nasoendoscopy recordings of the EPG task were used to extract still images of the pharyngeal constrictors at baseline “rest breathing” position and maximum pitch when PCmax occurred. First, the primary author underwent training by the senior author until reaching 90% or greater consensus on identifying still frames exhibiting the most representative rest position and PCmax for each participant. Each image was saved in .png format using VideoPad Editor (Version 8.16, NCH Software). Each rest and PCmax image within a participant were paired, coded, and inserted in a randomized sequence within PowerPoint, with 65% (30/46) of the pairs being presented twice to assess intrarater reliability. All image pairs were independently rated by judges blinded to a participant group to obtain interrater reliability estimates on 100% of image pairs. A total of 76 randomized image pairings were rated by judges.

### Judges

Two expert SLPs were recruited to provide ratings of PCmax. Each expert had a minimum of 3 years of experience dedicated toward voice assessment using nasoendoscopy. The judges conducted ratings of PCmax independently, unless consensus was necessary, as described below.

### Acoustic Signal Extraction

The audio recording from the nasoendoscopic recording was used to extract and measure audiovisual detection of voice onset to maximum fundamental frequency (*F*0) during the upward pitch glide. Identified segments were exported to a .wav format file using VideoPad Editor (Version 8.16, NCH Software). The .wav format file was imported into Praat (Version 2.0, 2015) for the measurement of the lowest and highest *F*0s. The lowest value was subtracted from the highest value to determine the maximum *F*0 range in Hz, which was converted to semitones (STs) to control for sex-based differences in pitch ranges measured in Hz. The maximum ST range was compared between groups; this comparison was conducted to evaluate whether primary dependent variable outcomes by etiology (UMN vs. LMN) could be explained by group differences in pitch range production.

### Nasoendoscopy Measures of PCmax

**Figure 4.**
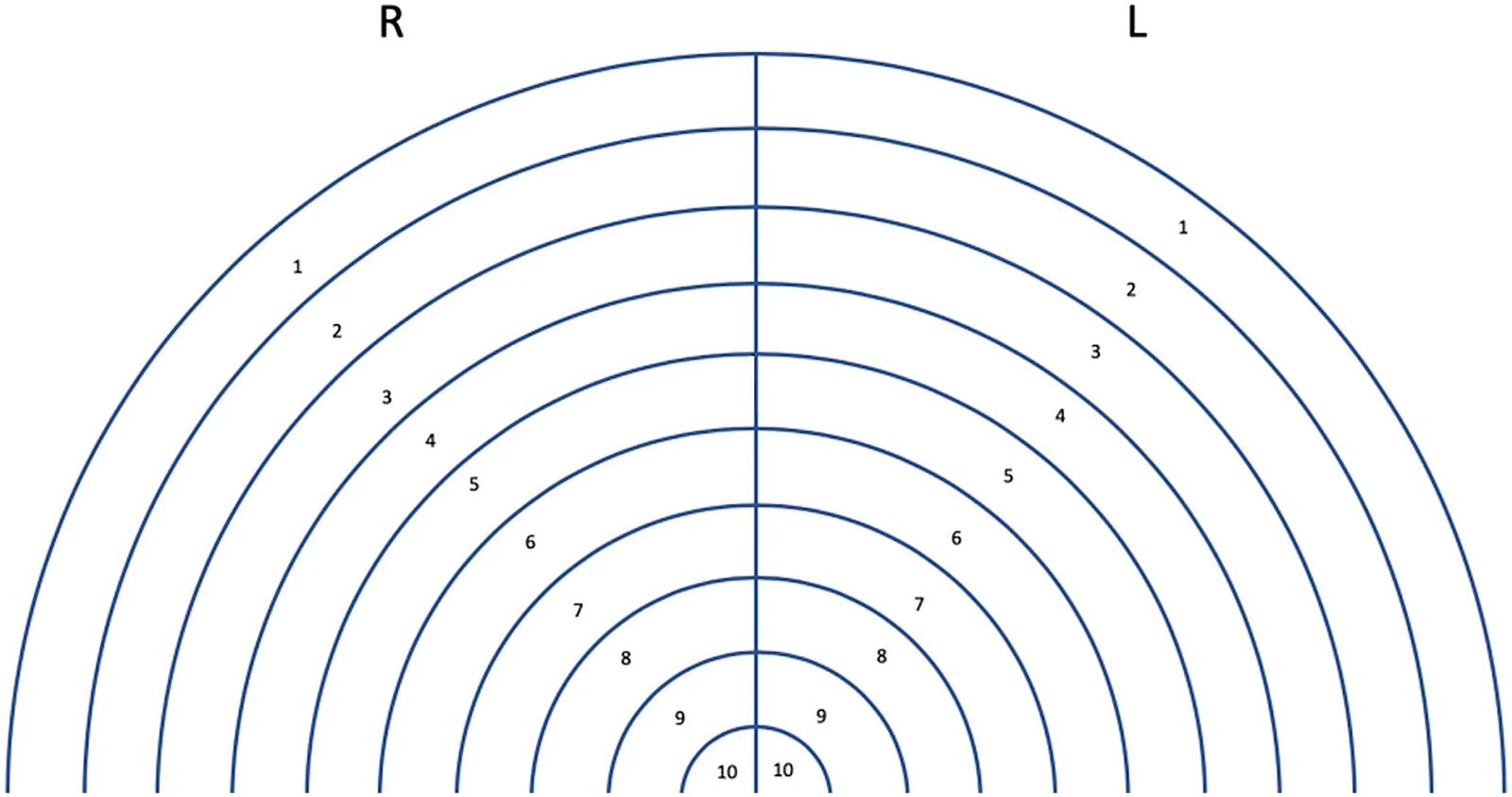
This figure shows the Pharyngeal Constriction Rating Form Scale used to measure maximum pharyngeal constriction (PCmax). R and L indicate the right (R) and left (L) sides of the pharynx. An absence of movement of the posterior pharyngeal wall would be indicated by a rating of 0. Constriction on each side of the pharynx was indicated by the line representing the closest degree to which the posterior pharyngeal wall reached with the maximum possible degree being 10 on each side. The rating for each side was summated for the measures of PCmax in this study.

Visual perceptual ratings of PCmax during the nasoendoscopy exam were completed using a pharyngeal constriction rating form created for this study (see Figure 4). Prior literature relied on binary (normal/abnormal) gross-level clinician judgments of pharyngeal constrictor function for comparison to VFSS outcomes (Fuller et al., 2009; Miles & Hunting, 2023). Two studies reported that rating the symmetry and degree of pharyngeal constriction during dynamic playback of the EPG was associated with poor clinician reliability (Fuller et al., 2009; Rodriguez et al., 2007). These authors found that requesting raters to perform binary judgments of normal/abnormal resulted in higher interrater reliability. However, details of symmetry and degree of pharyngeal constriction observed across patients was lost. In this study, we generated a visual perceptual rating form based on the regional air space of the pharynx occupied by the posterior pharyngeal constrictors and base of tongue/larynx at rest breathing posture. Instead of dynamic ratings, this form was created for raters to indicate the symmetry and degree of air space difference from rest breathing posture to PCmax observed between paired still images. Thus, the pharyngeal constriction rating form provided a consistent referent scale for comparing all visual perceptual ratings.

The pharyngeal constriction rating form displayed an equal-appearing interval spatial scale, with numbered segments representing the degree of PCmax observed on each side of the pharynx. The scale was created with a curved appearance based on the typical shape of the pharynx during rest. The scale enabled quantitative rating from 0 (*no constriction*) to 10 (*complete closure*) on the right and left sides of the pharynx. To represent the possibility of asymmetric versus symmetric constriction, a total score was recorded in addition to the individual ratings for each right (maximum of 10 points possible) and left (maximum of 10 points possible) pharyngeal side. Thus, a minimum score of 0 and a maximum score of 20 (with summation of the left and right side scores) were possible.

When comparing ratings between judges, a rating difference of “1” on one side of the pharynx was considered as equivalent. The exception to this was when a judge rated a “0” (no displacement) and another rated “1” (slight displacement). In this case, the judgment of “no movement” and “some movement” was considered a difference requiring consensus (described below) between judges. Similarly, participants with a rating that differed between judges by more than 1 for either side of the pharynx required consensus.

After both judges completed their independent ratings, items with the above-identified differences underwent discussion between the judges to achieve consensus. The final consensus rating was recorded as the final rating for discussed items. For all other ratings that met agreement criteria, the average rating from the two judges was recorded for the left and right pharyngeal ratings for the first paired image presented for each participant. Final left and right pharynx scores were summated to give a final measure of PCmax.

### VFSS Measures of Pharyngeal Constriction

VFSS recordings were acquired at 30 frames per second (fps) using TIMS software (TIMS Medical) and pulsed fluoroscopy at 25–30 pulses per second (depending on the year of the evaluation). Participants completed the protocol established by Leonard and Kendall (2025). This protocol was adopted at our clinic site in 2012 after Dr. Kendall joined the team and supported the implementation of the first published quantitative VFSS measurement approach associated with normative referent values. The Leonard and Kendall (2025) protocol includes the administration of two trials of 3-cc and 20-cc thin liquid barium sulfate contrast (Varibar). The smaller volume has been published as analogous to a typical secretion bolus volume during “dry” swallows, whereas the 20-cc bolus volume has been identified as the most challenging bolus to swallow; in addition, it elicits maximum pharyngeal constrictor activation and can be used to determine functionality of swallowing in individuals (Kendall et al., 2000; Leonard et al., 2000; Leonard & Kendall, 2025). Finally, only liquid boluses were studied to enable comparison of findings to prior literature investigating EPG correspondence with VFSS findings (Fuller et al., 2009; Miles & Hunting, 2023; Miloro et al., 2014).

During VFSS testing, individuals were given a medicine cup with liquid boluses to be swallowed and instructed to self-administer from the cup and swallow when ready. All participants were administered the 3-cc liquid bolus. If a participant exhibited silent aspiration, or the inability to expectorate aspirant of this bolus volume (Penetration Aspiration Scale Scores of 7–8), the 20-cc bolus was not administered by the clinician (Rosenbek et al., 1996). In addition, if a participant was unable to swallow the full 20-cc bolus (i.e., unable to initiate the swallow or was unable to swallow the entire cohesive volume at one time), testing of that bolus volume was terminated by the clinician.

Once VFSS recordings were obtained, they were saved as .avi files for measurement using either ImageJ (https://imagej.net/ij/) in combination with the Universal Desktop Ruler (http://avpsoft.com/products/udruler/), or Swallowtail software (Belldev Medical), depending on the year of the participant's assessment. Swallowtail was developed by Belldev Medical in collaboration with Drs. Leonard and Kendall, who validated that temporal and spatial measures derived using Swallowtail were comparable to those obtained using their previously established manual measurement methods, while allowing for more efficient analysis.

Measures of pharyngeal area at maximum constriction (PAM) were obtained from VFSS recordings performed as part of a standard clinical protocol based on previously published protocols and normative references by Drs. Leonard and Kendall. In brief, PAM is derived using a calibrated lateral view of each participant's VFSS recording during PCmax. Calibration of spatial measures is first conducted for each participant using image analysis software to measure the number of pixels represented by the known diameter of a dime taped to the chin of each recorded patient. Next, the frames showing the greatest degree of pharyngeal constriction during each participant's 3-cc and 20-cc swallow were identified. The SwallowTail software enables the clinician to trace and measure the pharyngeal area at maximum constriction (i.e., PAM) for comparison to previously published normative values from a group of age- and sex-matched healthy controls (Kendall et al., 2000, 2004; Leonard et al., 2000). Prior normative values were shown to differ by age (</≥ 65 years of age) and sex (male vs. female; Kendall et al., 2000, 2004; Leonard et al., 2000). To compare measures across participants, the severity of each participant's PAM measure was scored as “0” if it fell within the middle of normative measures, “1” if it was in the outer quartile, and “2” if it was outside the distribution of measures of age- and sex-matched healthy controls (Kendall et al., 2016).

The measurement protocol for PAM was conducted utilizing Swallowtail software to determine the pharyngeal area during PCmax during each bolus swallow (i.e., PAM; Leonard & Kendall, 2025). All clinicians in the clinic where measures were obtained are required to achieve an 80% or higher interrater agreement on the range of temporal and spatial measures documented (Ellerston et al., 2016).

Each participant's measurement of PAM was extracted from participant EMR documentation for the 3-cc and 20-cc liquid bolus conditions, apart from two subjects who did not complete the 20-cc bolus due to airway safety concerns after testing the 3-cc liquid bolus. Each PAM measure was compared to established normative ranges for the participant's age and sex (Kendall et al., 2000, 2004; Leonard & Kendall, 2025; Leonard et al., 2000). Due to the differing ranges of “normal” and “abnormal” values by age and sex, values were compared to the appropriate age and sex group and their measure was transformed into a severity score using an ordinal scale for analytic comparison as described earlier.

### Statistical Analysis

Demographics and clinical outcomes were summarized using mean, standard deviation (*SD*), median, and interquartile range. Wilcoxon rank-sum test and chi-squared or Fisher's exact test were used to determine whether the distribution of patients differed by demographic variables of age, sex, or medical diagnosis/history between LMN and UMN groups for continuous variables and categorical variables, respectively. A univariate logistic regression within group (i.e., UMN or LMN) was used to determine if the dichotomized version of summed degree of PCmax from nasoendoscopy ratings (normal vs. abnormal) associated with VFSS PAM measures. Intraclass correlation coefficient (ICC) estimates were calculated to determine the inter- and intrarater reliability of PCmax nasoendoscopy ratings. The ICC was interpreted using the method of Koo and Li (2016), with the lower bound of ICC 95% confidence interval (CI) less than 0.5 indicating poor reliability, values between 0.5 and 0.75 indicating moderate reliability, values between 0.75 and 0.9 indicating good reliability, and values greater than 0.9 indicating excellent reliability. Statistical analyses were implemented using R (Version 4.4.3, R Core Team, 2024).

## Results

### Participant Demographics

A total of 46 participants met inclusion criteria for this study, with 24 classified into the LMN and 22 in the UMN groups. As shown in Table 1, The UMN group was significantly older by ~10 years, on average, than the LMN group. Those in the LMN group averaged 63.2 years of age (±14.4 years), with 11 female and 13 male participants. The average age of those in the UMN group was 72.7 years (±10.9 years), with 10 female and 12 male participants. Most individuals in the LMN group had a medical history of unilateral vocal fold paralysis (54%), whereas the UMN group had equal proportions of individuals diagnosed with PD (52%) or stroke (48%). One member of the LMN group had a medical history of a brainstem-level (pontine) stroke resulting in LMN signs and symptoms in contrast to those in the UMN group with cortical and subcortical lesions.

**Table 1.** Subject demographics.

Variable	LMN (*n* = 24)	UMN (*n* = 22)	*p* value
Age (years)			
*M* (*SD*)	63.2 (14.4)	72.7 (10.9)	
*Mdn* (IQR)	64 (57.0, 68.5)	73 (67.0, 81.5)	.014
Range	23, 93	47, 86	
Gender			.59
Male	13 (56.5%)	12 (52.2%)	
Female	11 (52.4%)	10 (47.6%)	
Diagnosis			< .001
Stroke	1 (4.2%)	10 (48%)	
Parkinson's disease	0 (0%)	11 (48%)	
Status post frontal meningioma surgery	0 (0%)	1 (4%)	
Unilateral vocal fold paralysis	12 (50%)	0 (0%)	
Anterior cervical disk fusion (ACDF)	8 (33.3%)	0 (0%)	
Base of skull tumor surgery	2 (8.3%)	0 (0%)	
Idiopathic SLN paresis	1 (4.2%)	0 (0%)	
Language spoken			N/A
English	23 (95.8%)	22 (100%)	
Korean	1 (4.2%)	0 (0%)	
Time between exams (days)			
*M* (*SD*)	25.7 (18.5)	17.9 (19.3)	N/A
*Mdn* (IQR)	21 (15)	12 (24)	
Range (min, max)	69 (1, 70)	78 (0, 78)	

*Note.* Subject demographics and etiology of pharyngeal affect in lower motor neuron (LMN) versus upper motor neuron (UMN) lesion groups. SLN = superior laryngeal nerve; min = minimum; max = maximum; N/A = not applicable.

### Nasoendoscopy Rating Inter- and Intrarater Reliability

Intrarater reliability on nasoendoscopy PCmax ratings was considered moderate to good across raters. Rater 1 achieved a good ICC rating of .76 (95% CI [.63, .84]), while Rater 2 achieved a moderate rating of .71 (95% CI [.56, .82]). A moderate degree of interrater reliability of .73 (95% CI [.57, .82]) was achieved for nasoendoscopy PCmax ratings prior to consensus.

### VFSS and Nasoendoscopy PCmax Measures by Group

The odds ratio of higher PCmax scores corresponding with normal VFSS PAM scores was 1.91 (95% CI [1.14, 4.52], *p* = .053) in the LMN group for the 20-cc bolus volume and 2.2 (95% CI [1.25, 5.72], *p* = .032) for the 3-cc volume. No significant correspondence between PCmax and PAM occurred for the UMN group (*p* = .89, 20-cc bolus; *p* = .97, 3-cc bolus).

**Figure 5.**
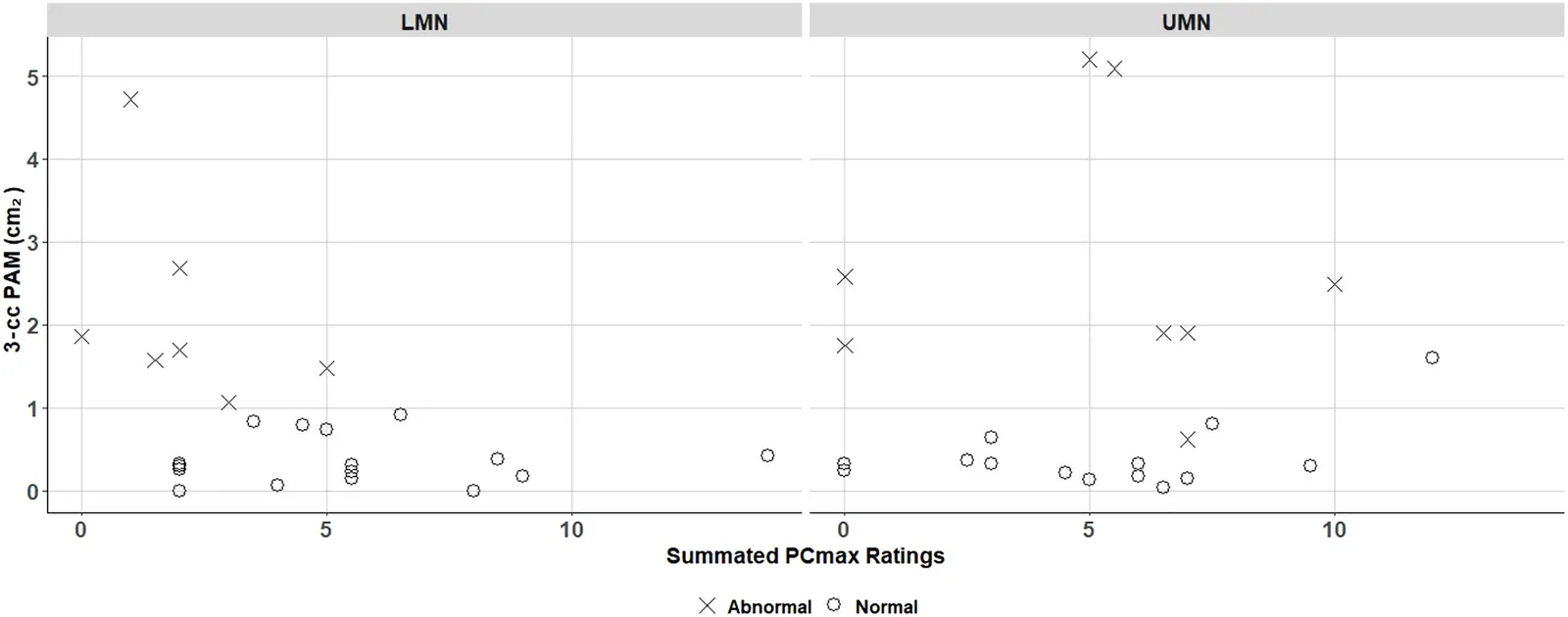
Summated maximum pharyngeal constriction (PCmax) ratings are plotted against 3-cc PAM scores in both the lower motor neuron (LMN) and upper motor neuron (UMN) groups. An abnormal PAM is indicated by “X,” whereas normal values are indicated by “O.” Note that there are no abnormal PAM scores above a summated rating of 5 in the LMN group.

**Figure 6.**
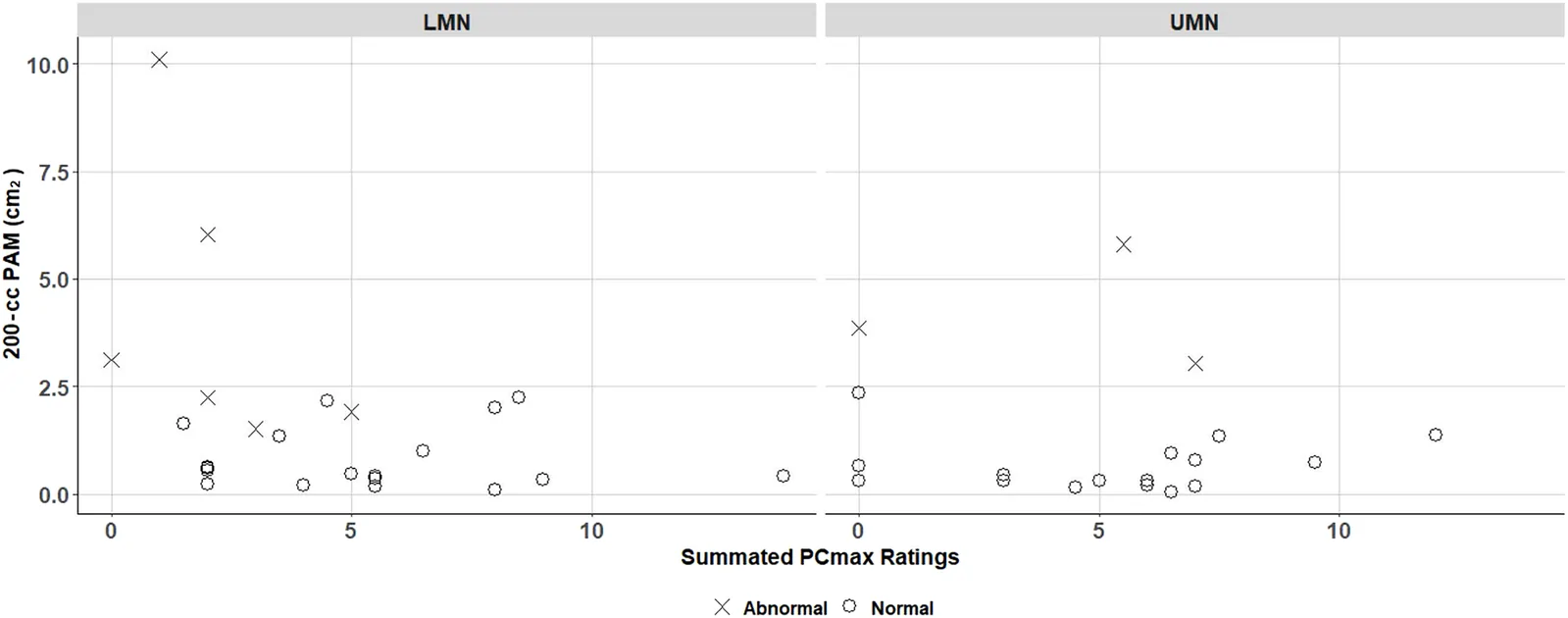
Summated maximum pharyngeal constriction (PCmax) ratings are plotted against 20-cc PAM scores in both the lower motor neuron (LMN) and upper motor neuron (UMN) groups. An abnormal PAM is indicated by “X,” whereas normal values are indicated by “O.” Note that there are no abnormal PAM scores above a summated rating of 5 in the LMN group.

Qualitative analysis of the summated PCmax scores identified that a summated score of 5 or greater corresponded with normal PAM measures in the LMN group regardless of bolus size (see Figures 5 and 6). In contrast, there were no predictive PCmax values found to correspond with predictable normal PAM values in the UMN group.

### Maximum ST Range by Group

Group-based comparisons of maximum ST range during the upward EPG task did not differ by group (see Table 2). This finding suggests that differences between PCmax and VFSS PAM in the UMN group were not associated with maximum pitch range group differences.

**Table 2.** Pitch range outcomes.

Outcome	Variable	UMN	LMN	*p* value
*F*0 range in ST: overall	*M* (*SD*)	17 (7.4)	16 (8.7)	.75
	*Mdn* (IQR)	17 (12, 21)	16 (9.4, 23)	
	Range	(3.6, 31)	(0.72, 31)	

*Note.* Pitch range outcomes. Descriptive statistics of the pitch range in semitones (STs). The distribution was found not to be distributed significantly differently between groups. UMN = upper motor neuron; LMN = lower motor neuron; *F*0 = fundamental frequency.

## Discussion

The purpose of this study was to evaluate whether PCmax measures obtained during a volitional task (i.e., EPG) corresponded to VFSS PAM measures during the nonvolitional patterned response swallow in those classified as having a UMN and LMN disorders. Outcomes of this study supported our hypothesis that PCmax and VFSS PAM measures would correspond highly for those in the LMN group. In addition, outcomes lend support to our hypothesis that PCmax measures obtained from nasoendoscopy would not reliably correspond with VFSS PAM measures in those with UMN lesions. Approximately 1/2 (9 to 12 of 22) of the UMN group showed poor correspondence between PCmax and PAM measures. During 3-cc bolus swallows, two UMN participants diagnosed with right hemisphere cerebrovascular accident and PD exhibited abnormal VFSS PAM measures, although their PCmax ratings indicated bilateral pharyngeal constriction. Three UMN participants showed similar outcomes across bolus volumes (see Table 3 for outcome by diagnosis), of which one notably had a summated PCmax rating of 10 (the second highest rating overall). Conversely, six UMN participants showed the opposite result. That is, abnormal PCmax corresponded with normal VFSS PAM outcomes (one was abnormal only during the 20-cc bolus, one was abnormal only during the 3-cc bolus, all others were abnormal during both the 3-cc and 20-cc boluses).

**Table 3.** Upper motor neuron (UMN) subjects with poor intertask correspondence by diagnosis.

Diagnosis	Summated rating	3-cc PCmax	20-cc PAM
Right hemisphere CVA	7.0	Abnormal	Normal
Left basal ganglia and left frontal cortex CVA	10	Abnormal	Abnormal
Left cortical CVA	0	Abnormal	Normal
Basal ganglia stroke	2.5	Normal	Abnormal^a^
Lacunar stroke	0	Normal	Normal
Parkinson's disease	6.5	Abnormal	Normal
Parkinson's disease	7.0	Abnormal	Abnormal
Parkinson's disease	3	Normal	Normal
Parkinson's disease	3	Normal	Normal
Parkinson's disease	0	Normal	Normal
Status post frontal meningioma removal	5.5	Abnormal	Abnormal

*Note.* UMN subjects with poor correspondence of intertask pharyngeal constriction, by diagnosis (increased summated rating should be associated with “normal” VFSS PAM). A summated score of 5 or higher corresponded with normal swallow outcomes in the LMN control group; therefore, a value above 5 could be expected to align with “normal.” Summated rating refers to the total (left and right) maximum pharyngeal constriction (PCmax) rating score, but it does not indicate bilateral versus unilateral constriction. In other words, a patient with unilateral pharyngeal constriction of “7” would receive the same score as a patient with bilateral activation of “3” and “4.” PAM outcomes are defined as normal or abnormal according to preestablished norms discussed above. CVA = cerebrovascular accident.

a Denotes a participant considered abnormal because 3-cc bolus findings were concerning enough for airway safety as to forego testing of the 20-cc volume.

These findings suggest that some individuals with impaired cortical or subcortical pathways exhibit variable recruitment of pharyngeal constrictor musculature during volitional voicing and nonvolitional patterned response swallowing. Thus, nasoendoscopic assessment of pharyngeal behavior during both EPG and swallowing is recommended in those with UMN etiologies to improve accurate clinical conclusions about pharyngeal constrictor function across tasks. That is, an EPG task alone may not consistently predict pharyngeal constrictor function during swallowing in those with UMN lesions.

Interestingly, approximately 50% of both groups (*n* = 23) exhibited asymmetrical pharyngeal constriction during EPG. In each group, nine individuals were judged to exhibit greater constriction of the left than the right pharynx. Only two individuals in the UMN and three individuals in the LMN groups were judged to exhibit greater constriction of the right than the left pharynx. However, asymmetry of pharyngeal constriction did not appear predictive of abnormal VFSS PCmax abnormalities in either group. Only five of the 12 individuals rated with poor correspondence between PCmax and PAM had asymmetric pharyngeal constriction. One individual was rated with a PCmax of 5.5 for the left side and 1.0 for the right side and achieved a normal VFSS PAM measure. Thus, asymmetric pharyngeal constriction during EPG may not predict abnormal PAM, although absence of movement during EPG was associated with abnormal VFSS PAM measures.

The PCmax ratings in this study differed from prior literature in that a referent rating scale based on visual perceptual ratings of the degree and symmetry of PCmax between paired still images of “rest breathing” posture and maximum pitch production. Prior work relied on binary clinical visual perceptual judgments (i.e., normal vs. abnormal) after reviewing dynamic EPG video recordings (Fuller et al., 2009; Kennedy et al., 2020; Rodriguez et al., 2007). These studies did not evaluate symmetry or scaled degrees of pharyngeal constriction. One prior study determined that interrater reliability was poor when judging pharyngeal symmetry or degree of constriction. To improve interrater reliability, the binary rating of normal or abnormal pharyngeal constriction was used which increases rater reliability to at least chance level (Rodriguez et al., 2007). In our study, raters achieved a moderate to good level of reliability, with several paired comparisons requiring consensus for final data analysis. Future work should evaluate whether adding referent anchors for the extreme and mid-levels of the scale for degree of bilateral and asymmetrical constriction would improve rater reliability similar to other nasoendoscopy rating scales (Lowell et al., 2017). It is also possible that advancements in semi- and automated approaches to image analysis may improve extraction of pharyngeal constrictor movements from endoscopic images to achieve higher consistency in measurement acquisition.

The rating form utilized in this study revealed interesting findings regarding pharyngeal constrictor patterns across LMN and UMN groups. For example, the summated value of “20” indicative of full bilateral pharyngeal closure was not achieved by participants in this study. The average summated PCmax score was 4.9 (*SD* = 3.3, *Mdn* = 5.0), with only three participants scoring a summated rating of 10 or greater. In addition, a summated PCmax score greater than 5 was associated with normal PAM scores in the LMN group (see Figures 5 and 6). Future investigation of PCmax outcomes in neurotypical adults is needed to evaluate normative reference values. Such information would enable comparison between this study's PCmax outcomes in UMN and LMN populations for improved interpretation of the finding that the summated PCmax score of 5 was associated with normal PAM measures in the LMN group. It is possible that those with LMN disorders achieved functional swallowing and normal PCmax measures while generating lower pharyngeal constriction swallow pressures than neurotypical populations (Galek et al., 2021; Jones et al., 2024). However, future work is needed comparing neurotypical, LMN, and UMN populations across PCmax and VFSS PAM measures, as well as pharyngeal pressure measures associated with typical versus impaired swallowing physiology (Galek et al., 2021; Jones et al., 2024).

Finally, EPG ST maximum ranges were compared between groups. This comparison was conducted to evaluate the possibility that UMN and LMN group differences in PCmax may have been influenced by the maximum pitch ranges performed by participants. However, comparisons of EPG maximum ST ranges were not significantly different between groups indicating comparable EPG maximum pitch ranges between groups. This was a surprising finding given that the UMN group (median age = 73 years) was significantly older than the LMN group (median age = 64 years). Literature has shown that maximum total pitch ranges progressively reduce after 60 years of age, giving reason to believe that the UMN group should have exhibited reduced ST range values compared to the LMN group (Knuijt et al., 2019; Konomi et al., 2016). Alternatively, the majority of the LMN group was composed of individuals with vocal fold paralysis, which can also reduce maximum pitch range performance due to impaired vocal fold muscle tone, either unilaterally or bilaterally (Konomi et al., 2016). It is possible that age- and disease-related factors offset group-based maximum ST range comparisons in this study. Future work comparing UMN and LMN groups on EPG and PSM should strive toward evaluation of age and sex influence on measured outcomes in addition to expanded consideration of other LMN etiologies as well as more homogenous UMN groupings controlling for lesion location and extent, to the degree possible. A prospective research design may be necessary to improve control over inclusion/exclusion group criteria, clinical testing timeline and implementation of clinical assessment protocols across participants to reduce variability introduced by reliance on clinical retrospective data collection.

Another potential limitation of our study was the omission of scaling factors for participant body size that is incorporated into a more recent quantitative methodology, Analysis of Swallowing Physiology Events, Kinematics, and Timing for use in Clinical Practice (Steele et al., 2023). Consideration of sex, age, and body size factors in future work may help identify predictor variables that inform high-risk patients with UMN likely to exhibit discrepancies between volitional voicing tasks compared to nonvolitional patterned response swallowing based on EPG and PSM maneuvers.

Another limitation to this study was the reliance on retrospective clinical data as well as inclusion criteria that resulted in a small participant population for comparison. Future studies conducted using a prospective research design could evaluate larger sample sizes with greater control over group-based inclusion criteria to ensure that the findings on this smaller population are replicated.

## Conclusions

This study addressed measures of pharyngeal constriction (PCmax) during nasoendoscopy and EPG (a volitional voice task) corresponding with VFSS PAM measures during swallowing (a nonvolitional patterned response) in those with dysphagia etiologies classified as UMN or LMN. Outcomes showed that PCmax measures did not consistently correspond with VFSS PAM in approximately 1/2 of the UMN group, whereas the LMN group had high intertask correspondence. These findings lend support to our hypotheses regarding the contraindication of generalizing pharyngeal constrictor function during volitional and nonvolitional patterned response activities in those with UMN lesions. In those patients, our findings endorsed direct observation of pharyngeal constriction function during swallowing rather than inferring it from an EPG task during nasoendoscopy.

## Data Availability Statement

The data sets generated and analyzed during this study are available from the corresponding author on reasonable request.
